# Optimization and fabrication of programmable domains for soft magnetic robots: A review

**DOI:** 10.3389/frobt.2022.1040984

**Published:** 2022-11-24

**Authors:** Alistair Bacchetti, Peter Lloyd, Silvia Taccola, Evan Fakhoury, Sandy Cochran, Russell A. Harris, Pietro Valdastri, James H. Chandler

**Affiliations:** ^1^ Centre for Medical and Industrial Ultrasonics, James Watt School of Engineering, University of Glasgow, Glasgow, United Kingdom; ^2^ Science and Technologies of Robotics in Medicine Laboratory, School of Electronic and Electrical Engineering, Faculty of Engineering and Physical Sciences, University of Leeds, Leeds, United Kingdom; ^3^ Future Manufacturing Processes Research Group, University of Leeds, Leeds, United Kingdom; ^4^ Industrial and Mechanical Engineering Department, Lebanese American University, Byblos, Lebanon

**Keywords:** soft robots material and design, magnetic materials, magnetic soft robots, design optimization, advanced fabrication, bio-inspired (BI), programmable magnetic profiling

## Abstract

Driven by the aim of realizing functional robotic systems at the milli- and submillimetre scale for biomedical applications, the area of magnetically driven soft devices has received significant recent attention. This has resulted in a new generation of magnetically controlled soft robots with patterns of embedded, programmable domains throughout their structures. This type of programmable magnetic profiling equips magnetic soft robots with shape programmable memory and can be achieved through the distribution of discrete domains (voxels) with variable magnetic densities and magnetization directions. This approach has produced highly compliant, and often bio-inspired structures that are well suited to biomedical applications at small scales, including microfluidic transport and shape-forming surgical catheters. However, to unlock the full potential of magnetic soft robots with improved designs and control, significant challenges remain in their compositional optimization and fabrication. This review considers recent advances and challenges in the interlinked optimization and fabrication aspects of programmable domains within magnetic soft robots. Through a combination of improvements in the computational capacity of novel optimization methods with advances in the resolution, material selection and automation of existing and novel fabrication methods, significant further developments in programmable magnetic soft robots may be realized.

## Introduction

Programmable magnetic soft robots (MSRs) are typically formed through the combination of magnetic particles with soft elastomeric materials, and are controlled wirelessly *via* external actuating magnetic fields. Variation of the material distribution and magnetic profile has enabled MSRs to demonstrate shape-forming and navigational abilities far beyond those of current soft robots (Zhang J. et al., 2021). Their potential for highly compliant shape-forming, wireless actuation and miniaturization to the milli- and submillimetre scales (Ju et al., 2021) presents MSRs as candidates well suited to minimally invasive medical and surgical applications, including for cell-based microgripper manipulation (Xu et al., 2019), shape-forming surgical catheters (Wang et al., 2021; Pittiglio et al., 2022) and origami-inspired micromachines for remote drug delivery (Cui et al., 2019).

**FIGURE 1:**
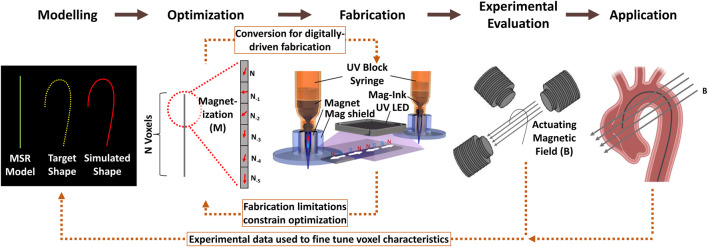
Idealized approach for voxel encoding and programmable MSR development. The presented example involves a voxelated biomedical soft magnetic continuum robot for cardiac catheterization. In modelling, the relevant target deflection path is extracted from patient data and modelled *via* finite element software. The model along with target deflection requirements are utilized by an integrated optimization protocol (e.g., a genetic algorithm) to determine voxel characteristics such as distribution, scale and magnetization. An automated, digitally-driven fabrication protocol (e.g. DIW) simultaneously fabricates and magnetizes 3D voxels throughout the structures in accordance with optimized data. The fabricated MSR is actuated and controlled in experiment *via* actuating magnetic fields. Experimental data may be integrated with the modelling and optimization protocol to fine tune voxel characteristics throughout further MSR design iterations. When an optimal shape forming configuration is achieved, the MSR may be deployed for application-specific evaluation. The critical bi-directions links between optimization and fabrication are highlighted. “Fabrication” subfigure adapted from Ma et al. (2021).

An idealized approach to the development of programmable MSRs is illustrated in Figure 1. In this scheme, high fidelity modelling of the MSR facilitates implementation of a requirements-driven design optimization based on a specific desired shape or application. Optimized magnetic profiles can subsequently be fabricated to encode spatially distributed magnetic domains (voxels) throughout the MSR geometry, and the resultant device experimentally verified and deployed. The application of actuating magnetic fields, applied throughout both experimental evaluation and application, are essential in facilitating the feedback of real experimental data for the fine tuning of programmable voxel characteristics. Although each aspect is critical in the overall capability of the resulting MSR, for voxel-based designs, interaction between optimization and fabrication often forms the critical limiting step.

This review aims to highlight the most significant developments and challenges specific to the optimization and fabrication of programmable domains for biomedical MSR applications. Following a review of programmable MSRs presented in the literature, we first consider specifically recent developments in optimization procedures, including physics-based modelling, deep learning and genetic algorithm protocols. This is followed by examination of novel fabrication methods across lithographic patterning, direct ink writing and direct laser writing that both support fabrication of optimal designs and limit optimization flexibility. We conclude the review with a discussion focused on the main current challenges and future directions of optimization and fabrication methods for the advancement of programmable MSRs.

### Programmable magnetic soft robot designs

Many MSR designs rely on magnetic profiling approaches, as depicted in Figure 1, which involves the encoding of specific magnetization strengths and directions throughout magnetically active geometries to achieve specific shape-forming configurations during actuation. Table 1 presents a summary of the most recent programmable MSR developments.

**TABLE 1:** Significant programmable MSR developments for biomedical application. Includes numerous bio-inspired designs. Summarizes developments, fabrication methods, optimization and modelling methods, approximate scales and sample applications of MSRs within key and supporting literature. A dash under ‘Optimization and Modelling Methods’ indicates that no methods were sourced in literature, or reported to have been developed, applied or investigated with respect to the highlighted MSR design.

MSR design	Optimization and modelling methods	Fabrication method	Approximate scale (mm)	Sample application
Modular MSR with multimodal locomotion Dong et al. (2022)	—	Template-assisted lithography	50–100	Remote therapeutic drug delivery for gastric ulcer treatment
Blood vessel anchoring machine Zhang et al. (2021a)	—	Soft lithography with jig-assisted assembly	0.50–1	Remote stem cell delivery for vascular tissue regeneration
Peristaltic pump device Zhang et al. (2021a) See Figure 2A	—	Soft lithography with jig-assisted assembly	1–1.50	Remote therapeutic drug delivery
Wireless soft capsule Zhang et al. (2021a)	—	Soft lithography with jig-assisted assembly	0.50–1	Remote therapeutic drug delivery
Minimally invasive continuum MSR Wang et al. (2021)	Genetic algorithm with finite element modelling	Doped silicone moulding	0.50–0.80	Minimally invasive cardiovascular navigation
Shape-forming tentacle MSR Lloyd et al. (2020); Pittiglio et al. (2022)	Artificial neural network with FEM modelling	Doped silicone moulding	30–40	Minimally invasive *in vivo* navigation
MSR with inchworm-inspired locomotion Wu et al. (2020)	Evolutionary algorithm with FEM modelling	Direct ink writing	15–20	Minimally invasive *in vivo* navigation
MSRs with earthworm, inchworm and pill bug-inspired locomotion Deng et al. (2020)	Predictive deformation analysis with FEM modelling	Direct laser writing	5–8	Minimally invasive *in vivo* navigation
Origami-inspired micromachine Cui et al. (2019) See Figure 2B	—	Soft electron beam lithography	0.02–0.10	Nanonewton-scale cell manipulation
Undulatory spermatozoid swimmer robots Xu et al. (2019)	Euler-Bernoulli beam modelling	Digital light processing lithography	1–5	Minimally invasive *in vivo* navigation
Six-arm microgripper robot Xu et al. (2019) See Figure 2C	Euler-Bernoulli beam modelling	Digital light processing lithography	1–2	Remote therapeutic drug delivery
Origami-inspired magnetic membranes Li et al. (2019)	—	Thermal curing	0.50–1	Implantable cell manipulation
MSR with jellyfish-inspired locomotion Hu et al. (2018)	Predictive deformation analysis with planar beam deformation modelling	Doped silicone moulding	1–1.50	Ultrasound-guided gastrointestinal navigation
MSR with caterpillar-inspired locomotion Hu et al. (2018)	Predictive deformation modelling with planar beam deformation modelling	Doped silicone moulding	1–1.50	Navigation throughout complex undulatory environments
Hexagonally voxelated 3D MSR Kim et al. (2018)	Predictive deformation analysis with FEM modelling	Direct ink writing	10–20	Remote therapeutic drug delivery
MSRs with jellyfish, spermatozoid and cilia-inspired undulatory locomotion Lum et al. (2016)	Predictive deformation analysis with planar beam deformation modelling	Doped silicone moulding	1–10	Minimally invasive *in vivo* navigation
2D magnetically active hydrogels Tasoglu et al. (2013)	—	UV-curable photolithography	0.50–1	Growth factor delivery for cell microenvironment manipulation

Early research into MSR profiling was largely limited to non-programmable (i.e., soft magnetic) materials with heterogeneous magnetic distributions, typically restricting shape-forming and navigational abilities to planar deformations (Xu et al., 2019). More recently, programmable (i.e., hard magnetic material) magnetic profiling (PMP) has been accompanied by the use of voxels. Voxel-based fabrication involves the patterned embedding of magnetic particles within an elastomeric polymer matrix (Kim et al., 2018; Xu et al., 2019; Bira et al., 2020), and commonly employs hard magnetic particles which retain a degree of magnetization after exposure to a strong magnetic field (Qiji et al., 2020; Macrae Montgomery et al., 2021; Zhao et al., 2021). Early work in the area of PMP was focused on scales of 100–500 µm and limited to domain encoding for 2D microstructures. Lee et al. (2010) developed 2D magnetic barcodes with sequences of superparamagnetic microparticles. Magnetic anisotropies were encoded *via* UV curing, which fixed characteristic magnetization properties to individual target domains. Kim et al. (2011) also developed a sequential magnetization process for superparamagnetic voxels, forming a conformable serpentine microactuator with variable magnetic responses and geometric deformations along its length.

**FIGURE 2:**
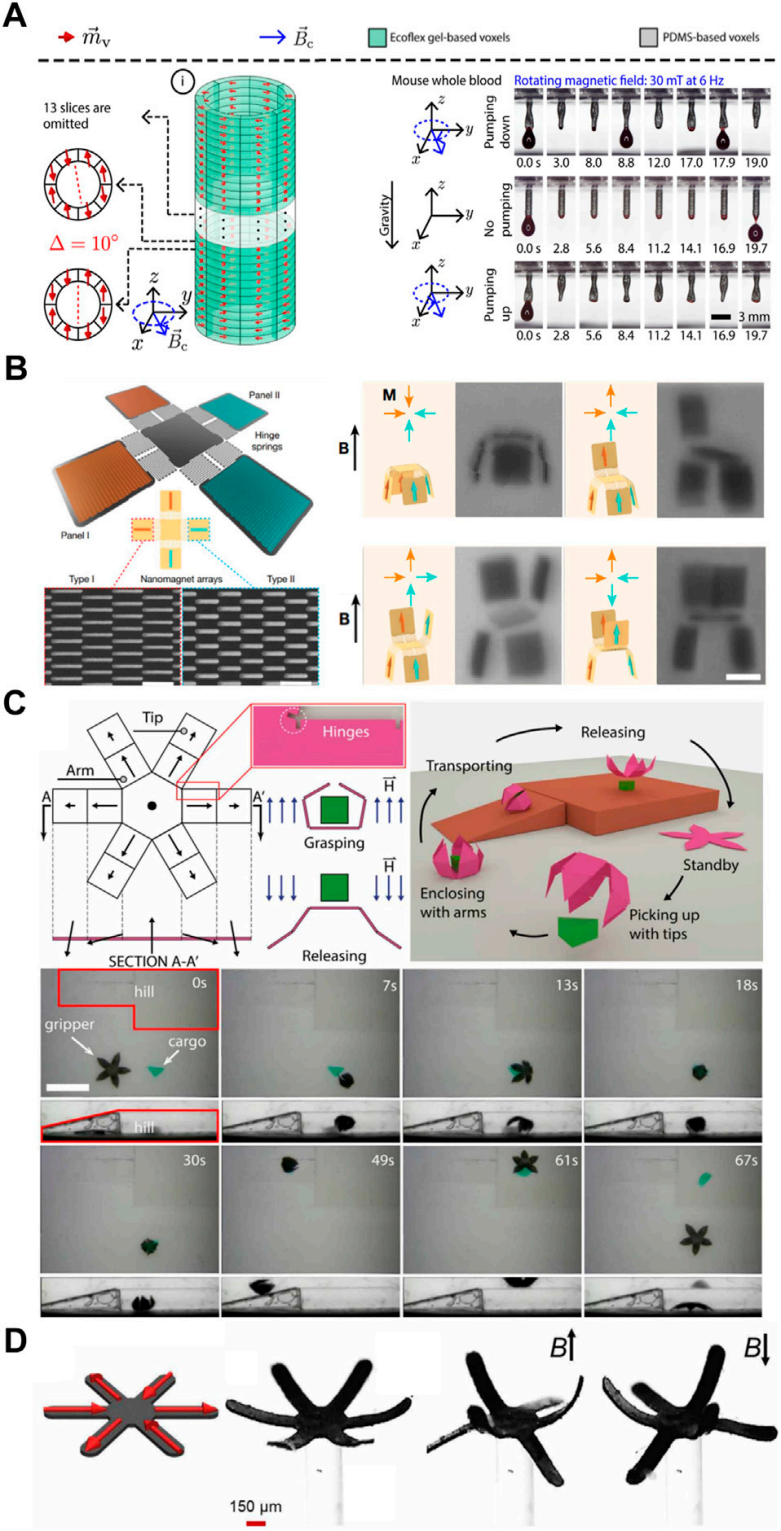
Examples of programmable voxel-based magnetic soft robots, demonstrating their structure and actuation **(A)** Magnetic microtubular soft robot observing wireless peristaltic actuation to pump microfluidic volumes of mouse blood from an attached reservoir (scale bar, 3 mm); from (Zhang J. et al., 2021), reprinted with permission from AAAS **(B)** Origami-inspired magnetic micromachine, observing multiple folded panel configurations for proposed nanonewton-scale cell manipulation (scale bars, 500 nm for the magnified nanomagnetic array image, and 10 µm for the shape configuration images); from (Cui et al., 2019), reprinted by permission from (Cui et al., 2019); Copyright ^©^ 2019 Springer Nature **(C)** Five-arm magnetic microgripper, observing sequential pickup, transport and release function for micrometre-scale cargo delivery (scale bar, 5 mm); from (Xu et al., 2019), reprinted with permission from AAAS **(D)** Six-arm magnetic microgripper, from observing reversable shape configurations in the direction of the applied magnetic field (scale bar, 150 µm); from (Alapan et al., 2020) under CC BY-NC 4.0.

Coupling voxel-based PMP approaches with specific designs allows MSRs to be selectively shaped under actuating fields. Resulting structures have demonstrated complex auxetic behaviours (Kim et al., 2018), undulatory locomotion (Diller et al., 2014; Bira et al., 2020), intricate shape-forming (Alapan et al., 2020) and self-folding abilities (Dong et al., 2019; Southern et al., 2019). A resurgence of interest in PMP has occurred in recent years, with many researchers seeking to leverage PMP to create programmable MSRs for a range of biomedical applications (Figure 2). Examples include voxelated tubular MSRs for microfluidic transport, developed by Zhang J. et al. (2021), which are capable of acting as a peristaltic pump when subjected to a rotating magnetic field; allowing transport of microvolumes of mouse blood and demonstrating its potential for wireless microfluidic transport within remote *in vivo* environments such as the endometrium (Figure 2A). Microgripper-based developments for nanonewton-scale cell manipulation (Cui et al., 2019), (Figure 2B), and micrometre-scale therapeutic drug delivery (Xu et al., 2019), (Figure 2C), have also been presented in recent years.

As highlighted in Figure 1; Table 1, MSR development using voxel-based designs is often reliant on interaction between optimization and fabrication. Despite recent advancements in these areas, significant challenges still present hurdles for programmable MSRs (Xu et al., 2019). Regardless of the scope of current optimization procedures, realising voxel miniaturization to the submillimetre scale and below may largely be limited by the associated MSR fabrication process. This increases the difficulty in realizing optimized models as physical miniaturized structures (Zhang J. et al., 2021). Furthermore, several fabrication methods consist of manual fabrication and assembly steps, restricting the process’ overall efficiency and potential for integration with iterative computational optimization methods. Lastly, the resolution of current fabrication methods is also restricted by the size of embedded magnetic particles, ranging from 5 to 50 µm in scale and a limited diversity of materials, hindering the potential of MSRs for many applications (Kuang et al., 2021).

## Advancements in programmable domain optimization

The aim of a successful programmable MSR optimization method is to integrate model-based results with the fabrication procedure to derive desired voxel distribution, scales and magnetization properties throughout MSR geometry. The integration of computational optimization methods with current fabrication techniques presents a significant hurdle to future programmable MSR development. This is particularly prominent at submillimetre voxel scales, whereby greater computational power is required as greater densities of individual voxel characteristics must be solved with respect to global actuation behaviour. However, multiple developments in the optimization of programmable domains have been presented, as outlined in this section.

### Optimization *via* physics-based modelling

Modelling can be achieved through analytical, numerical or data-driven methods, and provides a predictive foundation upon which both voxel and external control field properties can be evaluated. Physics-based modelling allows for the simulation and visualization of programmable MSR behaviours alongside a range of variable parameters, including magnetic field strength, geometrical shape and magnetic particle concentration (Fernandez-Pacheco et al., 2017; Lloyd et al., 2020; Chunping et al., 2022). Controlled variables within these representative models of deformable MSR behaviour can then be optimized for specific desired outcomes, often comparing the MSR’s actuated behaviours against the anatomical geometries of the target tissue, such as a blood vessel or convoluted surface. This provides a physics-based foundation upon which targeted voxel design and magnetization parameters may be based to optimize MSR deformation.

Xu et al. (2019) utilized a physics-based model to determine small voxel deformations within programmable MSR sheets. These visualizations were integrated with a custom MATLAB (The Mathworks, Cambridge, United Kingdom) script which divided and simulated models as voxelated elements through Euler-Bernoulli beam modelling. This method verified large angle deformations throughout programmable MSR geometry. When integrated with digital light processing lithography, the model informed the optimized design of several parameters for a bio-inspired undulatory swimmer robot, including tunable swimming speed. Deng et al. (2020) also developed a physics-based model to inform the design and distribution of magnetic anisotropies throughout pill bug-inspired MSRs. The model simulated and visualized the deformation behaviour of each configurable MSR film under an applied magnetic field.

Current challenges of physics-based modelling often involve computational limits, particularly when considering submillimetre voxel resolutions. The simultaneous modelling of large numbers of voxels may severely reduce overall efficiency throughout the computational process, whilst increasing the risk of unsuccessful convergence within simulated models. Furthermore, models to date are typically under the simplifying assumption of static or quasi-static conditions and thus lack the possibility to capture critical dynamic aspects of MSRs under actuation. Assumptions also extend to material considerations, where in appropriate considerations of hyper-elastic material properties and deformable magnetic domains can lead to potentially significant output errors (Di Lecce et al., 2022). Another key challenge remains in capturing the MSR’s underlying physics at high fidelity and across a range of voxel scales. This includes interpreting viscous and frictional forces and self-interaction between magnetic regions at submillimetre scales. It is therefore pertinent to investigate methods with reduced computational burden and integration with realtime predictive model data, such that programmable MSR behaviours may be optimized with ever-increasing accuracy and reliability (Byoungkwon et al., 2018).

### Optimization *via* deep learning algorithms

Deep learning optimization integrates statistical modelling with iterative neural network computation to derive voxel configurations for desired deformation. The integration of deep learning algorithms with MSR fabrication has potential to significantly improve the selectivity and computational reach of MSR optimization, in turn paving the way for a new generation of optimized programmable MSRs.

Lloyd et al. (2020) utilized an artificial neural network optimization protocol, which received input magnetization information alongside iterations of desired FEM results for the rapid prototyping of millimetre-scale continuum MSRs. Development of the artificial neural network allowed for the processing of high volumes of simulation data, reducing the overall manual input required for experimental analysis. This allowed for rapid realization of optimal shape configurations and desired tentacle actuation behaviour.

Despite the capacity and increasing popularity of deep learning algorithms, such methods cannot be easily generalized beyond the specific training set in question. Furthermore, acquisition of data must be performed either *via* experiment, which is arduous, or *via* simulation, which reintroduces the aforementioned errors in modelling assumptions. This presents a significant challenge to adoption in deep learning-based optimization of programmable magnetic domains (Lloyd et al., 2020).

### Optimization *via* genetic algorithms

Optimization *via* genetic algorithms involves the stochastic sampling, replication and transfer of successful voxel characteristics to further design iterations, yielding MSRs with highly optimized physical properties such as workspace size, localized magnetizations and rigidity patterns (Wang et al., 2021). This approach holds the advantages of a theoretically unlimited search space (no entrapment in local minima) and can be applied to non-differentiable functions, including model-free experimental arrangements (Whitley, 1994). However, it is more computationally intense than deep learning methods.

Wang et al. (2021) utilized a genetic algorithm which maximized the physical workspaces of continuum MSRs through iterative identification of optimal rigidity and magnetization properties in 3D space. This yielded MSR tips with conformable properties and strong agreement with associated modelling results, demonstrating the capacity of genetic algorithm integration for optimized bio-inspired design. Wu et al. (2020) utilized an evolutionary algorithm to achieve desired deformations of cantilever MSR sheets. A three-layered voxel structure with seven encoding patterns was used to form the algorithm’s initial genotype. The algorithm utilized target deformation data from real time FEM to program tunable magnetization densities and directions for genotype encoding. This could generate complex parabolic, cosine and half-circle deformation shapes for inchworm-inspired locomotion.

Current challenges lie in the integration of genetic algorithms with a wider variety of voxel fabrication methods. This is due to current generic algorithm protocols typically being bespoke and interlinked with a particular voxel-based design and fabrication method, developed and utilized by a single research group such as that of Wang et al. (2021). However, through additional research, genetic algorithm protocols may be improved to facilitate generalized programmable MSR optimization across a wider variety of high-resolution, digitally-driven voxel-based fabrication methods.

## Advances in programmable domain fabrication

The aim of a successful programmable MSR fabrication process is to deliver appropriate material distribution of magnetic and non-magnetic elements in the form of high-resolution voxels, and in optimized arrangements as determined by the interlinked optimization protocol. Beyond the base requirements, it is advantageous to facilitate fabrication across scales and with a wide variety of compatible materials. Realising these elements in a single, automated platform represents a significant challenge; however, numerous manual and digitally-driven approaches to the fabrication of programmable magnetic domains have been proposed, as outlined below.

### Lithographic patterning

In terms of programmable MSR fabrication, LP comprises a family of additive methods which involve embedding magnetic particles within curable substrates, followed by selective magnetization and composite curing (*via* molds or templates) to impart varied magnetic properties between cured layers. LP realizes exceptional applicability for single and multidirectional voxel magnetization, ranging from binary magnetization patterning in 1D to arbitrary multi-layer encoding in 3D programmable materials. It has therefore played an integral role, alongside studies into magnetic, complex 3D shape configurations, for biomedical MSR prototypes (Zhang J. et al., 2021).

A digital light processing lithographic method was developed by Xu et al. (2019) which imparted localized magnetic anisotropies to hard magnetic nanoparticle domains *via* selective curing of a UV resin. Embedded particles were first magnetized with an external magnetic field. A template was then utilized to expose particular composite regions to UV light, locking in the embedded particle magnetizations. This automated process was used to fabricate 2D elastomeric MSRs with programmable magnetic anisotropies and facilitated the development of a bio-inspired undulatory swimming robot which utilized encoded travelling wave motion along its length to achieve flagellar motion, commonly observed in spermatozoa and bacterial cells. This may hold promising applications for minimally invasive navigation throughout complex vasculature or cerebral spinal fluid (Manamanchaiyaporn et al., 2020).

In contrast, Zhang et al. (2021) utilized a soft lithographic method to batch-fabricate voxelated 3D structures with auxetic properties which showed shrinkable deformations when placed under rotating magnetic fields. The method tuned magnetic anisotropies to resolutions of 35 μm, allowing for the precise encoding of individual UV-curable voxels throughout 3D MSR geometry. In a separate manual step, voxels were then assembled with non-magnetic silicone moulded jig faces, which connected and oriented adjacent voxels for desired magnetization characteristics. The method was utilized to fabricate, encode and assemble the voxelated tubular MSRs for microfluidic transport. However, each step of this process was performed manually, which reduced efficiency of the overall fabrication process.

A lithographic approach was also taken by Dong et al. (2022), but based on a template-assisted method. This utilized highly specific templates to transfer prescribed magnetic microparticle patterns and magnetization profiles onto double-sided layers of adhesive PEI tape. Compared to the step-by-step fabrication and assembly process of Zhang et al., Dong et al. used double-sided adhesive tape to permit a simpler manual process by eliminating the additional requirement of individual domain assembly. However, even in this case, the assembly step was largely non-automated. This process was utilized to fabricate a multimodal programmable MSR with potential for remote therapeutic drug delivery for gastric ulcer treatment.

Current challenges for LP voxel-encoding lie in the limited availability of biocompatible materials (Xu et al., 2019). Future research may address this by integrating gelatin or hydrogel-based materials (with more readily tuneable mechanical properties), (Xu et al., 2019; Zhang J. et al., 2021), with hard magnetic particles for programmable shape-forming (Zhang S. et al, 2021). Coupling these materials with the high specificity of LP may allow fabrication of highly programmable, bio-inspired MSRs with sufficient biocompatibility or even biodegradability for safe *in vivo* application (Erb et al., 2012; Gao et al., 2016).

### Direct ink writing

DIW couples digitally-driven methods of fused deposition modelling with programmable magnetization to produce voxelated structures with readily encoded magnetic anisotropies (Wu et al., 2020). DIW incorporates embodied, selective deposition throughout the MSR’s fabrication process, often through the application of a variable magnetic field at the ink extruder nozzle for rapid programming of a magnetically active ink. This suggests exceptional potential for an efficient, integrated extrusion and magnetization method with far shorter fabrication times than soft lithography (Kim et al., 2018; Wu et al., 2020).

Kim et al. (2018) utilized a customized DIW method, involving an ink mixture of neodymium-iron-boron and fumed silica particles, embedded within a silicone matrix. The development of a support ink allowed for the fabrication of auxetic 3D structures with sufficient structural stability and multi-layer support throughout extrusion. This method was used to design hexagonally voxelated 3D MSRs with readily programmed magnetic anisotropies at millimetre scales. Under a magnetic field, the MSRs could be curled up and rolled along a surface. Optimizing such behaviour could fine-tune the devices for wireless, controllable delivery of therapeutic drugs to remote areas of the body. Furthermore, integration with biodegradable inks may allow for the complete degradation of the programmable structure, reducing the requirement of device retrieval following remote drug delivery.

A similar method was developed by Wu et al. (2020) who applied a longitudinal magnetic field near the extruder nozzle to magnetize the ink’s embedded particles along the specified printing direction. Through precise printing path control, multi-layer linear MSR structures with pre-programmed voxel magnetizations were fabricated for reconfigurable shape-forming. This method was integrated with an evolutionary algorithm design protocol, allowing for the rapid encoding of pre-defined curvatures along the MSR’s length. This was demonstrated through the development of a programmable inchworm-inspired MSR, which performed biomimetic crawling through the encoding of maximum deformations at the robot’s midpoint, and minimal deformations at each end.

At present, DIW suffers from deteriorating print quality at submillimetre scales, largely as the scale of embedded magnetic particles is approached. Additionally, shorter voxel lengths at the submillimetre scale induces weaker magnetic densities between adjacent voxels. Both of these issues reduce the MSR’s overall sensitivity to external magnetic actuation, as well as its ability to achieve deformations of greater complexity (Wu et al., 2020). Future developments may seek to integrate DIW with alternative, higher-resolution fabrication procedures, including LP methods. Furthermore, integration with algorithm-based methods may allow for the rapid encoding of patient-specific anatomical geometries and desired deformations into individual voxels.

### Direct laser writing

Direct laser writing couples digitally-driven laser curing with programmable magnetization to selectively encode magnetic anisotropies at submillimetre scales. Similar to previous methods, DLW involves embedding magnetic particles within curable elastomeric matrices, often *via* optical additive manufacturing. A magnetic field programs anisotropies throughout each printed voxel layer, whilst a laser scans across target regions for localized encoding of magnetic anisotropies. The application of heat by laser scanning prevents thermal diffusion of magnetic particles to non-target regions, whilst rapid cooling fixes the selected magnetization patterns before consecutive layers are printed. The integration of DLW with pre-existing fabrication methods presents promising opportunities for the rapid, automated fabrication of programmable domains at micrometre scales (Deng et al., 2020; Li and Diller, 2022).

Deng et al. (2020) demonstrated significant progress through their development of a DLW-integrated soft lithographic procedure. This allowed for the continuous encoding of complex 2D MSR films at resolutions of 0.30 mm. This was used to encode rolling pill bug motion in a kirigami-inspired MSR film. The MSR’s magnetization directions were directed towards a single centreline along the film’s midpoint, instructing the voxels to fold up and roll forwards under a rotating magnetic field. The method’s combination of layer-by-layer encoding with additive fabrication presents opportunities in expanding to the 3D domain and investigating bio-inspired MSRs for customizable, patient-tailored treatments.

Alapan et al. (2020) also developed a novel DLW method which reprogrammed voxel domains at high spatial resolutions. This utilized laser heating to orientate and programme specified magnetic anisotropies within target voxels of multicomponent 2D MSR structures. Its exceptional resolution range allowed for whole-structure programming *via* global laser heating and individual voxel-programming at resolutions of 30 µm. The method was used to encode various magnetization profiles on a flower-inspired six-arm gripper robot (Figure 2D). The six-arm gripper could be exceptionally useful in single-cell anchoring and collection methods within the human body, developments which may reduce reliance on invasive cell collection methods for disease screening. The method could also be used to selectively reconfigure each petal’s magnetization pattern. This represents a significant step change in the encoding of programmable domains, for wireless, rapid reconfiguration of localized magnetic robots.

Currently, limitations in laser penetration depth remain a significant challenge in DLW, in turn limiting the method’s expansion to rapid 3D fabrication and programming (Alapan et al., 2020). Future developments may seek to improve the spatial selectivity of current scanning techniques for improved optical penetration and spatial encoding in 3D structures. Furthermore, future research may seek to integrate biocompatible hydrogel-based polymers with improved DLW methods (Sydney Gladman et al., 2016). This was recently demonstrated by Nojoomi et al. (2018), who utilized a heating method to encode deformation patterns in thermally responsive, biocompatible hydrogel sheets.

## Other fabrication methods

Other fabrication methods, including injection molding and magnetic spraying, have also played an essential role in the development of programmable MSRs for biomedical applications. Low-pressure injection molding methods for soft continuum MSR fabrication have recently been presented (Lloyd et al., 2020). Lloyd et al. (2020) injection moulded magnetically doped silicone to fabricate conformable magnetic tentacles, for experimental validation and optimization of magnetization properties alongside their previously described artificial neural network. Pittiglio et al. (2022) also developed a sequential low-pressure silicone injection molding procedure which embedded a series of cylindrical, programmable magnetic regions within a conformable silicone matrix, forming optimizable magnetic catheters which observed follow-the-leader motion for bronchial navigation. A similar method was developed by Wang et al. (2022), who embedded a series of programmable magnetic cubes within an injection moulded, biodegradable mixture of glycerol, gelatin and water to produce shape-forming, serpentine robots for wireless *in vivo* drug delivery. To support flexible, rapid fabrication of MSRs, Yang et al. (2020) developed a novel adhesive magnetic spray method, involving the application of a biodegradable PVA-based magnetic spray to a millimetre-scale, shape-forming catheter and ellipsoidal pill capsule for minimally invasive navigation and drug delivery within the gastrointestinal tract.

## Discussion and conclusion

It is evident that, even within a short period, significant developments in the optimization and fabrication of programmable domains have played an integral role in miniaturizing and improving the functionality of bio-inspired MSRs. As indicated in Table 1, the diversity of approaches coupling recent optimization and fabrication advancements has produced numerous miniaturized programmable MSRs with unprecedented capabilities in magnetic shape-forming and programmability.

As outlined, the field holds exceptional potential *via* the integration of reliable and computationally efficient optimization procedures with flexible fabrication methods. To maximize this potential, it is therefore pertinent to address the resolution, material, computational and automation challenges that affect the presented optimization and fabrication methods.

The computational capacity of physics-based modelling remains a significant hurdle in determining optimal voxel behaviours at submillimetre scales and across the full spectrum of underlying physical interactions which influence programmable MSR actuation. With the identified need for improved models to capture the dynamics, material properties and interaction forces, the required current and potential computational burden of physics-based modelling is significant. The integration of real data to tune adaptive models may be pursued to reduce the computational burden of future physics-based model methods. In deep learning methods, the trade-off between experimental data and simulation data use remains a significant challenge to widespread adoption. Efforts to reduce modelling assumption errors, particularly within underlying FEM models, may be applied for improved reliability of simulation-driven deep learning methods. The choice to utilise experimental data within future optimization procedures may depend on the voxel scale and resolutions applied throughout MSR geometry, with submillimetre scales less likely to be optimized *via* experimental data integration due to difficulty of reliable data collection at such scales. At present, optimization *via* genetic algorithms remains the most promising method, largely due to its operation with model-free experimental arrangements, capability of unlimited search space sizes and potential for increased adoption across various digitally-driven programmable MSR fabrication methods.

Additionally, significant challenges in the resolution, material selection and automation of current fabrication techniques must also be addressed. The limited availability of biocompatible materials remains a significant hurdle for current LP methods, however, additional research into the use of gelatin and hydrogel-based materials may improve the method’s potential as a widely adopted programmable MSR fabrication procedure. Furthermore, several LP and other fabrication techniques comprise a series of manual steps, reducing the efficiency of the overall MSR fabrication process and hence limiting both its reproducibility and scalability for high-volume fabrication. These manual processes, however, bring greater flexibility for precise domain assembly, as observed in Zhang et al.’s (2021a) method. Such flexibility is more difficult to achieve with digitally-driven processes such as DLW, which adopt a more automated, high-resolution protocol for programmable magnetic profiling. Regarding DLW, resolution issues at the submillimetre scale continue to outweigh its potential for high-resolution MSR fabrication, largely due to deteriorating print qualities as the magnetic microparticle scale is reached. Integration with current high-resolution methods which can accommodate submillimetre scale fabrication, such as LP, alongside novel optimization methods such as genetic algorithm optimization, may improve the potential of DLW in fabricating programmable MSR structures at high voxel resolutions. At present, the poor penetration depths of DLW restrict its expansion to voxel encoding within 3D structures. However, developments to improve the spatial selectivity of the method’s laser-scanning protocols may improve its overall potential for deep optical penetration and voxel encoding in 3D programmable MSRs. New digitally-driven, computer-controlled processes hold great promise for greater compositional variation and control, and engineering across different scales. Additionally, their flexibility, repeatability and higher production speeds will be important to the industrial translation of these devices.

It is widely anticipated that improvements in the capacity of both statistical and physics-based optimization coupled with further improvements to the resolution, material selection and automation of existing and novel fabrication methods will pave the way for further novel programmable MSRs. If an appropriate voxel resolution can be achieved across both optimization and fabrication aspects of MSR development, extended shape-forming abilities and improved biocompatibility hold the potential to unlock novel applications at the milli- and submillimetre scales.
